# Where Electrostatics Matter: Bacterial Surface Neutralization and Membrane Disruption by Antimicrobial Peptides SAAP-148 and OP-145

**DOI:** 10.3390/biom12091252

**Published:** 2022-09-07

**Authors:** Djenana Vejzovic, Paulina Piller, Robert A. Cordfunke, Jan W. Drijfhout, Tobias Eisenberg, Karl Lohner, Nermina Malanovic

**Affiliations:** 1 Institute of Molecular Biosciences, University of Graz, Humboldtstrasse 50/III, 8010 Graz, Austria; 2 Department of Immunology, Leiden University Medical Center, 2300 ZA Leiden, The Netherlands; 3 Field of Excellence BioHealth, University of Graz, 8010 Graz, Austria; 4 Bio TechMed Graz, 8010 Graz, Austria

**Keywords:** electrostatic interaction, membrane activity, *E. coli*, *E. hirae*, lipid–peptide interaction

## Abstract

The need for alternative treatment of multi-drug-resistant bacteria led to the increased design of antimicrobial peptides (AMPs). AMPs exhibit a broad antimicrobial spectrum without a distinct preference for a specific species. Thus, their mechanism, disruption of fundamental barrier function by permeabilization of the bacterial cytoplasmic membrane is considered to be rather general and less likely related to antimicrobial resistance. Of all physico-chemical properties of AMPs, their positive charge seems to be crucial for their interaction with negatively charged bacterial membranes. Therefore, we elucidate the role of electrostatic interaction on bacterial surface neutralization and on membrane disruption potential of two potent antimicrobial peptides, namely, OP-145 and SAAP-148. Experiments were performed on *Escherichia coli*, a Gram-negative bacterium, and *Enterococcus hirae*, a Gram-positive bacterium, as well as on their model membranes. Zeta potential measurements demonstrated that both peptides neutralized the surface charge of *E. coli* immediately after their exposure, but not of *E. hirae*. Second, peptides neutralized all model membranes, but failed to efficiently disrupt model membranes mimicking Gram-negative bacteria. This was further confirmed by flow cytometry showing reduced membrane permeability for SAAP-148 and the lack of OP-145 to permeabilize the *E. coli* membrane. As neutralization of *E. coli* surface charges was achieved before the cells were killed, we conclude that electrostatic forces are more important for actions on the surface of Gram-negative bacteria than on their cytoplasmic membranes.

## 1. Introduction

Rational peptide design is a challenging task in antimicrobial research. With the increasing need for alternative treatment of infectious diseases due to the increase in multidrug antibiotic resistance [1], there is a large increase in studies designing novel antimicrobial peptides (AMPs) [2]. The very promising feature of AMPs is their membrane-permeabilizing property [3,4,5,6,7,8], hence, they target the fundamental permeability barrier of pathogenic microorganisms, making resistance development less likely [9]. At the cellular level, AMPs damage the membrane to different extents. In many cases, a clear and fast membrane disruption is observed [8]. In some other cases, this process is slower and involves a number of sequential events including depolarization, destabilization, reorganization, and changes in the fluidity of the bacterial membrane leading to membrane permeabilization [10,11,12]. Although the mode of action of AMPs with respect to membrane disruption is considered to be a general phenomenon to a broad range of bacterial species, there is no clear consensus of efficient membrane disruption leading to bacterial cell death. Both, peptide properties and the composition of the bacterial membrane are known to strongly determine the antimicrobial activity of the peptides and thus, their mode of action [9,13].

**Figure 1:**
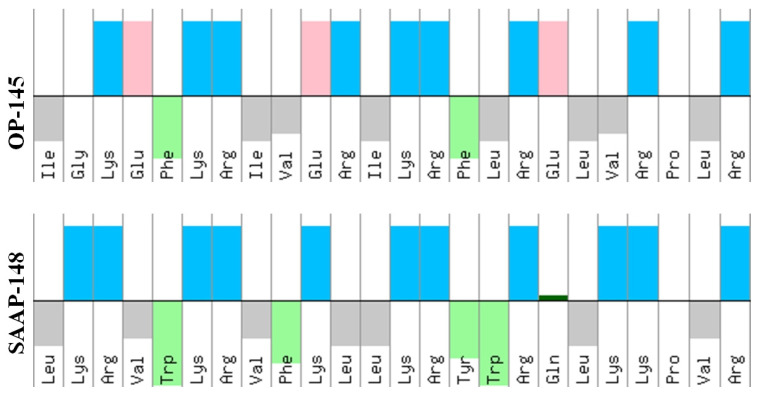
Peptide sequences. Distribution of amino acid residues in OP-145 and SAAP-148. Both peptides are N-terminally acetylated and C-terminally amidated, which is not shown in the pictures above. The hydrophilic residues are shown on top and hydrophobic on bottom as illustrated by Peptide Property Calculator (https://pepcalc.com/, accessed on 1 May 2022). Color code: acidic (red), aromatic (green), cationic (blue), aliphatic (grey), and polar (dark green).

In general, it is difficult to define key physico-chemical features of the peptides, that must be modified to improve their biological activities. Different strategies and peptide design principles for antimicrobial application have been developed [14]. Most modifications include major determinants of AMPs function, e.g., charge, hydrophobicity, amphipathicity, H-bonding capacity, conformational flexibility, and in vivo stability. Due to high levels of hydrophobic and cationic residues in AMPs [13], positive charges are believed to be responsible for the initial binding of the AMP to the negatively charged bacterial membrane, while hydrophobicity is proposed to dominate the insertion into and perturbation of the membrane [13,15,16,17]. These features (Figure 1) have also been studied in OP-145 [8,18,19,20], an AMP derived from the human cathelicidin LL-37 [21,22,23,24]. Like its parent peptide, OP-145 belongs to a class of linear cationic peptides, which adopts nearly an ideal alpha helix in presence of the membrane [20]. In buffer, it is largely unstructured; thus, the change in structural conformation seems to be a substantial part of the peptide’s interaction with the membrane. In further developments, all negatively charged amino acids in the OP-145 sequence were replaced and the number of positively charged amino acids was nearly doubled resulting in a peptide named SAAP-148 [8]. The overall hydrophobicity was not changed, but the hydrophobic character in the hydrophobic region was increased, while the predicted alpha-helical structure and total amphipathicity were kept almost constant [19]. Indeed, in our recent studies [8,19] we confirmed more efficient membrane disruption and an improved antimicrobial profile for SAAP-148. However, we could not observe the direct involvement of electrostatic interactions [19] to be essential for the disruption of model membranes composed of major bacterial phospholipids (PLs) identified in Gram-positive *E. hirae* [25]. Given the decrease in molar Gibbs energy of transfer from octanol into water ΔGW/OCT [26] for SAAP-148, the effects on membrane disruption could rather be attributed to more efficient insertion of the SAAP-148 into the membrane due to its larger hydrophobic area.

**Figure 2:**
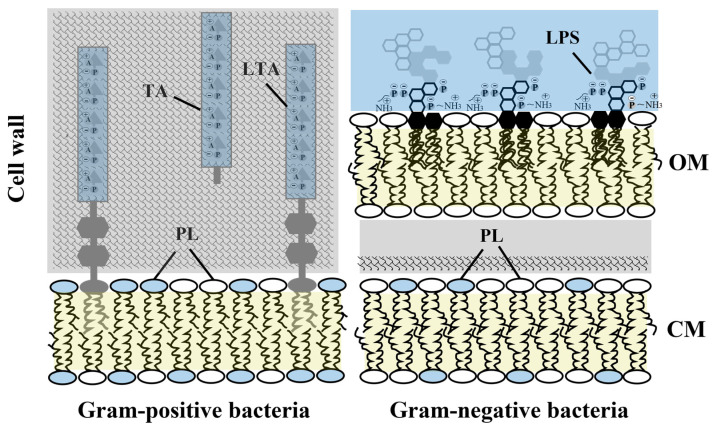
Cell envelopes of Gram-negative and Gram-positive bacteria. Negatively charged lipids distributed across the outer membrane (OM) and cytoplasmic membrane (CM) are shown in blue.

Our results [19] are very surprising because peptide affinity for anionic PLs is supposed to be crucial in determining the interactions between antimicrobial peptides and target membranes [13], and the cytoplasmic membrane of our model system, the Gram-positive bacteria *E. hirae*, consisted of solely anionic PLs [19]. However, there is also a large distribution of anionic PL between the bacterial species. Except for some narrow differences [13,27,28], cytoplasmic membranes of Gram-positive bacteria are composed of a high amount of negatively charged PLs. For instance, *E. hirae* cytoplasmic membrane [25] primarily consists of anionic phosphatidylglycerol (PG) and cardiolipin (CL), and to a lesser extent, phosphatidylethanolamine (PE) is present in *Bacillus subtilis* [29]. However, Gram-positive cytoplasmic membranes are still even more negatively charged than Gram-negative cytoplasmic membranes. *E. coli* cytoplasmic membranes consists of 75% zwitterionic phosphatidylethanolamine (PE) and 25% PG [9,13,30]. One of the aspects that is usually forgotten is that AMPs have to pass through the whole cell envelope, the cell wall of Gram-positive or the outer membrane of Gram-negative bacteria before being able to approach the cytoplasmic membrane [9]. The cytoplasmic membrane is not located on the surface of the bacteria and it is not the place of initial action of the AMP. AMPs are forced to interact with different targets of the cell envelope, which manage their attraction to bacterial surfaces. Hence, the surface of bacterial membranes is negatively charged because of the presence of large amounts of glycolipids such as lipopolysaccharides (LPS) in the outer membrane of Gram-negative bacteria and (lipo)-teichoic acids (LTA/TA) in Gram-positive bacteria [9] and not because of the presence of anionic phospholipids alone (bacterial membrane architectures showing potential interaction targets of the peptides in Figure 2).

In this study, we aim to get a deeper insight into which steps electrostatic forces are the leading driving force of the antimicrobial and membrane activity. For that, we tested OP-145 and SAAP-148 against one Gram-negative and one Gram-positive strain, *E. coli* versus *E. hirae*. Especially, by measuring the surface charge of those bacteria and their respective model membranes, we looked if peptides need electrostatic interactions to be attracted to the bacterial surface or for membrane disruption.

## 2. Materials and Methods

### 2.1. Materials

The phospholipids 1 palmitoyl-2 oleoyl-sn glycero-3 (phosphor-race-(1 glycerol)) (POPG, PG), 1 palmitoyl-2 oleoyl-sn glycero-3 phosphoethanolamine (POPE, PE), and polar *E. coli* lipid extract were purchased from Avanti Polar Lipids (Alabaster, AL, USA). Sodium chloride, chloroform, and methanol were obtained from Carl Roth (Graz, Austria) and 4-(2-Hydroxyethyl) piperazine-1 ethanesulfonic acid (HEPES, ≥99.5%) was from Sigma–Aldrich (Vienna, Austria). Unless otherwise indicated, all solutions were prepared in HEPES buffer pH 7.4, containing 10 mM HEPES and 140 mM NaCl or NaPi buffer containing 20 mM NaPi (Na_2_HPO_4_/NaH_2_PO_4_, 140 mM NaCl, pH = 7.4. ANTS (8-amino naphthalene-1,3,6-trisulfonic acid, disodium salt) and DPX (p-xylene-bis-pyridinium bromide) were from (Molecular Probes, Eugene, OR, USA) and propidium iodide (PI)was from (Invitrogen, Waltham, MA, USA) purchased from Thermo Fischer Scientific (Vienna, Austria).

### 2.2. Bacterial Strains

*Escherichia coli* ATCC25922 and *Enterococcus hirae* ATCC10541 were obtained from LGC Standards GmbH (Wesel, Germany).

### 2.3. Antimicrobial Peptides

The peptides OP-145 and SAAP-148 were synthesized as reported [8,20].

### 2.4. Growing Conditions of Bacterial Strains E. coli and E. hirae

For overnight cultures, Mueller–Hinton broth (MHB) and brain-heart infusion broth (BHIB) (Carl Roth, Graz, Austria) were inoculated with *E. coli* or *E. hirae* cells with a single colony placed at 37 °C under shaking conditions overnight. Thereafter, the main culture was inoculated starting at OD = 0.05 for *E. coli* and OD = 0.01 for *E. hirae* and incubated in fresh MHB/BHIB medium to mid-logarithmic phase for 3.5 h at 37 °C by shaking until the OD reached approximately 1.

### 2.5. Antimicrobial Activity of OP-145 and SAAP-148

After culturing the cells, the mid-log growing cells were washed once with NaPi buffer. Bacterial culture of 1 × 10^6^ CFU/mL *E. coli* and *E. hirae* in PBS or HEPES buffer were incubated for 5 min with the peptide concentrations of OP-145 and SAAP-148 ranging from 0.2 to 6.4 µM. Antimicrobial activity was then determined by plating a 100 µL sample on diagnostic sensitivity MBH/BHIB agar and subsequent establishing the number of viable bacteria after incubation at 37 °C overnight. The 99.9% lethal concentration (LC_99.9%)_ implies antimicrobial activity and constitutes the lowest peptide concentration that killed ≥99.9% of bacteria [2]. In analogy to zeta potential measurements, higher cell densities of 1 × 10^7^ CFU/mL *E. coli* were incubated for 5 min and *E. hirae* for 15 min with the peptides (0.8 µM–51.2 µM) in Hepes buffer.

### 2.6. Permeabilization of Bacterial Membranes

The cultures of 600–1200 µL 1 × 10^6^ CFU/mL cells previously washed with NaPi buffer twice (5 min, 5100× *g*) were supplemented with 1 µg/mL PI in polystyrene tubes. The fluorescence of PI was recorded by flow cytometer BD LSR Fortessa using the BD FACSDiva Software (Version 8.0.1, Beckton Dickinson, San Hose, CA, USA; excitation: 488 nm, emission: 695/40 nm, PerCP-Cy5.5 channel). Selective gating was applied to differentiate between PI-positive (permeabilized) and PI-negative (cells with intact membranes) cell populations. On average, 300–400 events per second were used for scan rate. Peptides at their lethal concentration LC_99.9%_ were added after 30 s to the PI-stained bacterial cells, and fluorescence was observed for 10 min for OP-145 and SAAP-148 treated cells. The FCS files obtained from the BD FACSDiva Software were converted into CSV files with the help of open-source FCSExtract Utility (version 1.02) and analyzed to calculate the percentage of PI-positive cells at different time intervals in Microsoft Excel.

### 2.7. Measurement of Zeta Potential of LUVs and Bacterial Strains

Zeta potential measurements were carried out according to the method described by Malanovic et al. [31] on a Zetasizer Nano (ZSP, Malvern Panalytical, Prager Electronics, Wolkersdorf, Austria) using disposable polycarbonate capillary cells (ZSP, Malvern Panalytical, Prager Electronics, Wolkersdorf, Austria). Large unilamellar vesicles (LUVs) were used in composition of POPG (PG), POPE/POPG (PE/PG = 3:1 mol) or *E. coli* polar lipid extract (PE/PG/CL = 67:23:10 mol). HEPES buffer was filtered using a syringe filter with 0.02 µm pores (Anotop, Sigma Aldrich, Vienna, Austria) and mixed with the 50 µM LUV suspensions and the appropriate peptide amount, 0.125–8 µM for membrane models and 0.8–51.2 µM for bacterial cells. For zeta potential measurements on bacteria, the cells at 1 × 10^7^ CFU/mL were mixed with filtered HEPES buffer followed by the addition of the peptide at various concentrations to 1 mL cells each. With the same instrument, the size of the membrane vesicles was measured.

### 2.8. Preparation of Liposomes

Membrane vesicles composed of POPG (PG), POPE/POPG (PE/PG = 3:1 mol), or *E. coli* polar lipid extract (PE/PG/CL = 67:23:10 mol) were prepared following the method described by Malanovic et al. [31]. Briefly, lipids were dissolved in chloroform/methanol, mixed to desired concentrations, and thin lipid films are obtained by evaporation under the stream of nitrogen and vacuum overnight. Then, the buffer of interest was added, and vesicles were formed under vigorous vortexing and 1 h incubation at 65 °C. LUVs were extruded through a 100 nm Whatman filter and their size was measured by Zetasizer Nano (ZSP, Malvern Panalytical, Prager Electronics, Wolkersdorf, Austria).

### 2.9. Permeabilization of Model Membranes/Vesicle Leakage Assay

For leakage experiments, LUVs composed of POPG (PG), POPE/POPG (PE/PG = 3:1 mol) or *E. coli* polar lipid extract (PE/PG/CL = 67:23:10 mol) were prepared in 10 mM HEPES buffer, pH 7.4 containing 68 mM NaCl, 12.5 mM ANTS, and 45 mM DPX. Leakage of the aqueous content of the 50 µM ANTS/DPX loaded lipid vesicles upon incubation with peptides of 0.125–8 µM was determined as described previously [31]. Fluorescence emission was recorded at 37 °C as a function of time before and after the addition of peptides. Triton X-100 was used as a positive control for maximum induced leakage of fluorescent dye.

## 3. Results

A concentration-dependent bacterial killing assay was performed first to determine the concentration range where peptides exhibit their antimicrobial activity. The concentration of the peptides necessary to kill 99.9% of *E. coli* and *E. hirae* cells within 5 min of incubation period was found to be 1.6 µM and 0.4 µM for SAAP-148 and 6.4 µM versus 3.2 µM for OP-145 when tested in NaPi buffer for 1 × 10^6^ CFU/mL bacteria (Table 1). These values are in accordance with previously published antimicrobial profiles of both peptides for a variety of Gram-negative and Gram-positive bacteria ([8,18,19]). Of note, higher concentrations of the peptides are necessary to kill both bacteria when tested in Hepes buffer and when higher cell densities of bacteria are used.

### 3.1. Antimicrobial Peptides Neutralize the Surface Charge of E. coli but Not of E. hirae

**Figure 3:**
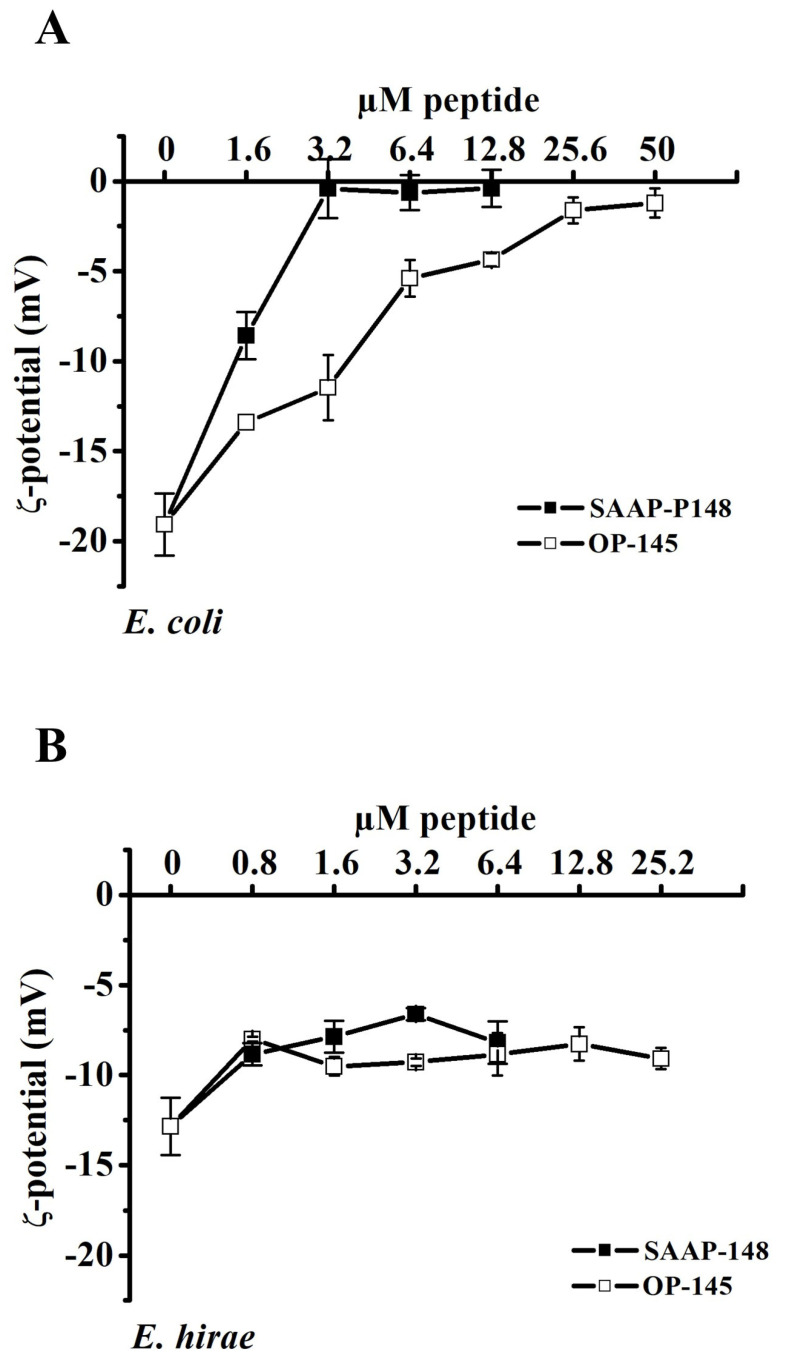
Zeta potential measurements of *E. coli* (**A**) and *E. hirae* (**B**) in the absence and presence of OP-145 (empty squares) and SAAP-148 (filled squared) at indicated concentration ranging from 0.8 to 51.2 µM. Data points are averages ± standard deviations of three independent experiments.

In order to study the effects of peptide binding on the surface charge of Gram-positive and Gram-negative bacteria, zeta potential measurements were performed on live cells. The bacterial surface charge was measured upon exposure to the peptides far below the killing concentration. This allows us to assess if electrostatic interaction or neutralization of the cell surface occurs before killing. The cell surface of *E. coli* exhibits a negative zeta potential close to −20 mV, which we observed to decrease in magnitude upon the addition of OP-145 or SAAP-148 (Figure 3A). Interestingly, 3.2 µM SAAP-148 was sufficient to neutralize the surface of *E. coli*, whereas 25.6 µM of OP-145 was needed to cause the same effect. As at those concentrations, the LC_99.9%_ was not reached (Table 1, LC_99.9%_ for 1 × 10^7^ CFU/mL, Hepes are performed in analogy to zeta potential experiments) we conclude that neutralization of the *E. coli* surface occurs during the killing process. By contrast, no major change in the surface charge of *E. hirae* could be observed (Figure 3B).

### 3.2. The Cytoplasmic Membrane Is Not Always a Direct Target Related to Cell Death

Both peptides are known to interact with bacterial lipids and permeabilize bacterial membranes [8,19]. To make a direct comparison between the peptide’s potential to permeabilize Gram-negative and Gram-positive membranes in relation to their lethal concentration, we performed flow cytometry. For this purpose, we used the membrane-impermeable DNA intercalating fluorescent dye propidium iodide (PI) as a marker for compromised membranes. PI enters the cell due to an abnormal, unspecific, and protein-independent increase in molecule flow across the disrupted cytoplasmic membrane.

**Figure 4:**
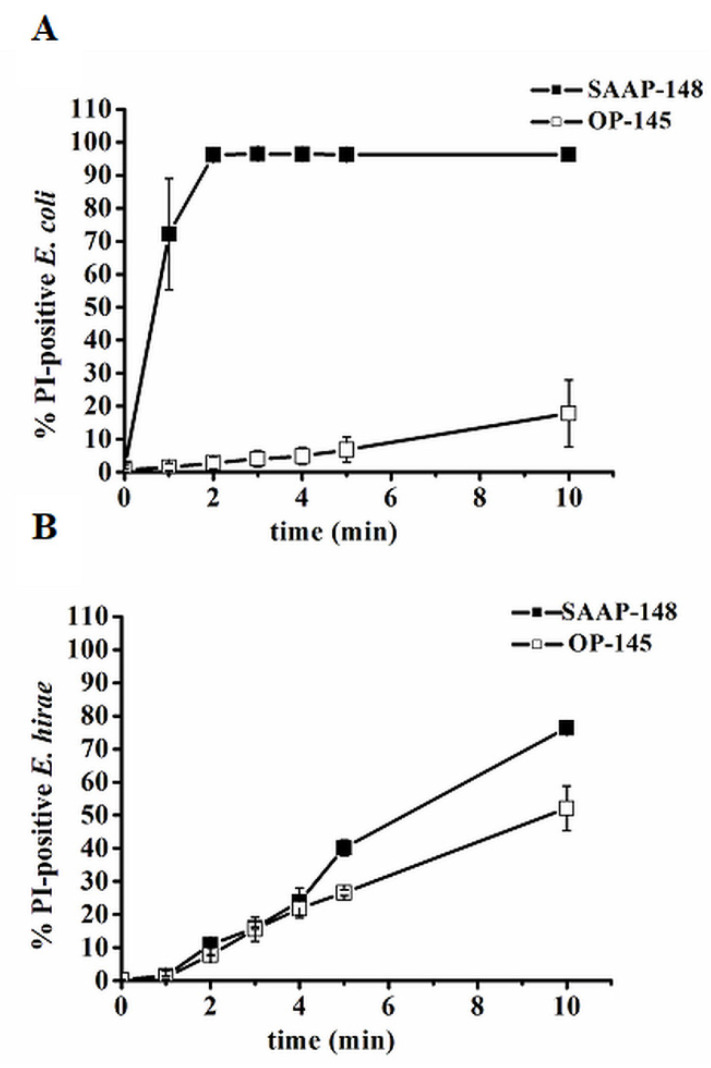
Membrane permeability of *E. coli* (**A**) and *E. hirae* (**B**) in absence and presence of OP-145 (empty squares) and SAAP-148 (filled squared) at concentration where they kill 99.9% cells. Data points are averages ± standard deviations of three independent experiments.

Peptides were applied to bacteria at a concentration where they kill bacteria within 5 min (Figure 4). Indeed, flow cytometry revealed an increase in PI-staining of *E. coli* (Figure 4A) and of *E. hirae* (Figure 4B) immediately after exposure to SAAP-148 pointing to substantial damage to the membrane. Whereas 70% of *E. coli* were already stained within the first minute, the *E. hirae* membrane reached this value first after 10 min of exposure to SAAP-148. Although OP-145 permeabilized the *E. hirae* membrane similarly to SAAP-148, this is not the case for OP-145 applied to *E. coli*. The number of PI-positive cells was negligible even after 10 min of incubation suggesting that the *E. coli* cytoplasmic membrane is not a direct target of OP-145.

### 3.3. Antimicrobial Peptides Neutralize the Surface Charge of Model Membranes

**Figure 5:**
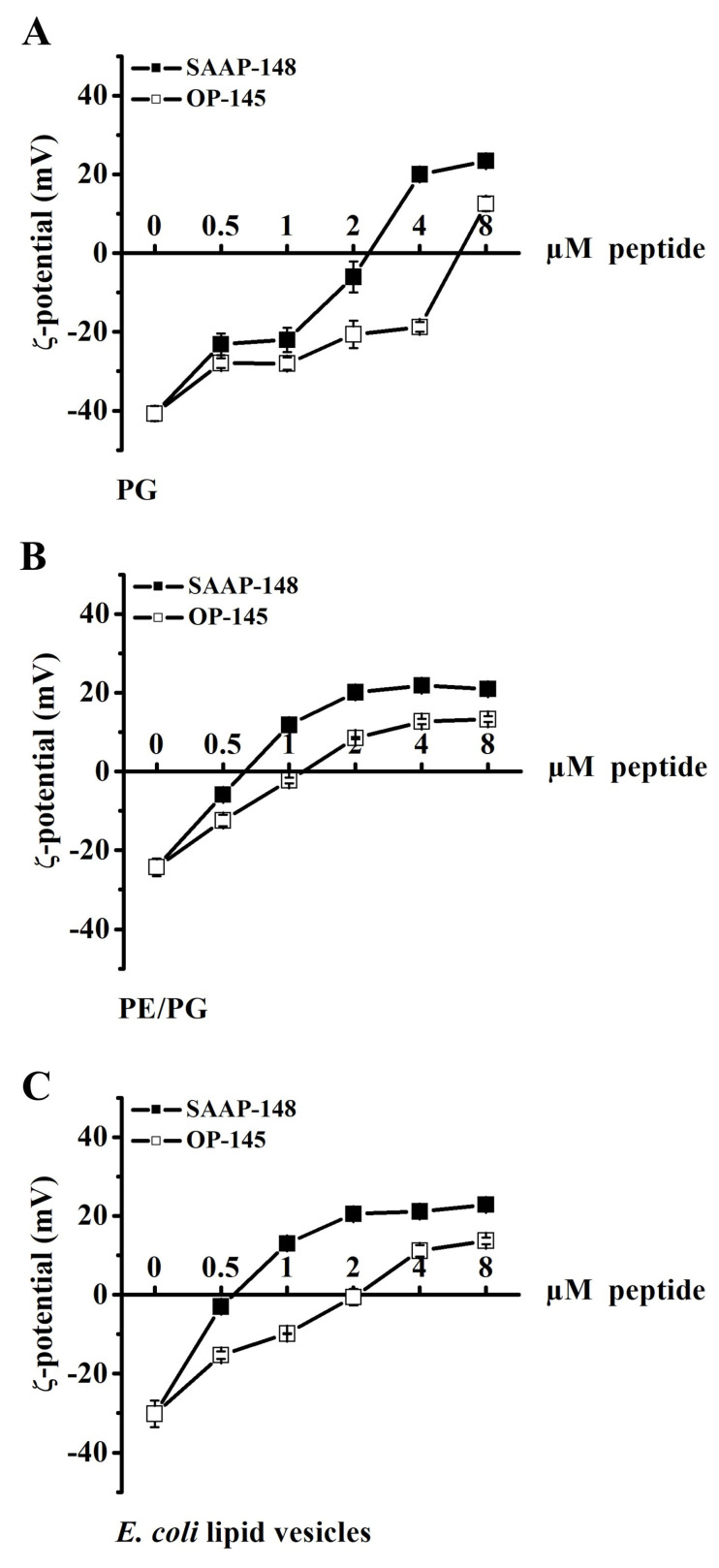
Zeta potential measurements of membranes PG (**A**), PE/PG (**B**), and *E. coli* polar lipids (**C**) in absence and presence of OP-145 (empty squares) and SAAP-148 (filled squares) at indicated concentration ranging from 0.5 to 8 µM for membrane model which corresponds to lipid to peptide molar ratio of 100:1-6.25:1. Data points are averages ± standard deviations of three independent experiments.

Next, we assessed zeta potential measurements on model membranes composed of solely anionic PG, partly anionic mixture PE/PG, and *E. coli* lipid vesicles (PE/PG/CL). Of note, measurements on PG/CL membranes resembling *E. hirae* cytoplasmic membrane recently published by our group [19] are performed in a similar fashion. All three model systems contain the most abundant phospholipids of bacterial membranes allowing indirect comparison with their biological counterparts. The experiment will show if the peptides are capable of neutralizing membrane surfaces composed of only phospholipids without interference from proteins or any other targets. As expected, results showed that both peptides neutralized all three membrane models. Interestingly, once neutralization of initially negatively charged lipid vesicles was reached, further addition of peptides resulted in overcompensation of the surface charge and a positive zeta potential (Figure 5). In our previous studies [20], we observed that upon exposure to OP-145 anionic vesicles aggregate to form cochleates in presence of divalent cations, but this was not observed on neutral vesicles and not under normal conditions in presence of monovalent ions. The measuring of the size in parallel to assay on anionic PG membranes (Figure 5) revealed that neither OP-145 nor SAAP-148 induced changes in vesicle size distribution during the interaction. This might suggest that overcompensation of the surface charge corresponds to the amount of the peptide remaining on the surface. Such charge reversal was not observed for live *E. coli* cells, where the zeta potential did not reach a positive value after neutralization (Figure 3A). Again, in all three model membranes, a higher concentration of OP-145 was needed to neutralize surface charge in comparison to SAAP-148. Further, a higher concentration of both peptides is needed to neutralize the surface charge of solely anionic PG membranes than that of partly anionic PE/PG or *E. coli* membrane models, respectively. SAAP-148 neutralizes PG membranes around 2 µM and others around 0.5 µM. A similar situation was found for OP-145; 8 µM OP-145 neutralized the surface of PG but much fewer peptides 1–2 µM are needed for the same effect in partly anionic membranes.

### 3.4. Antimicrobial Peptides Disrupt Efficiently Anionic Membranes, but Not Less Anionic Membranes

**Figure 6:**
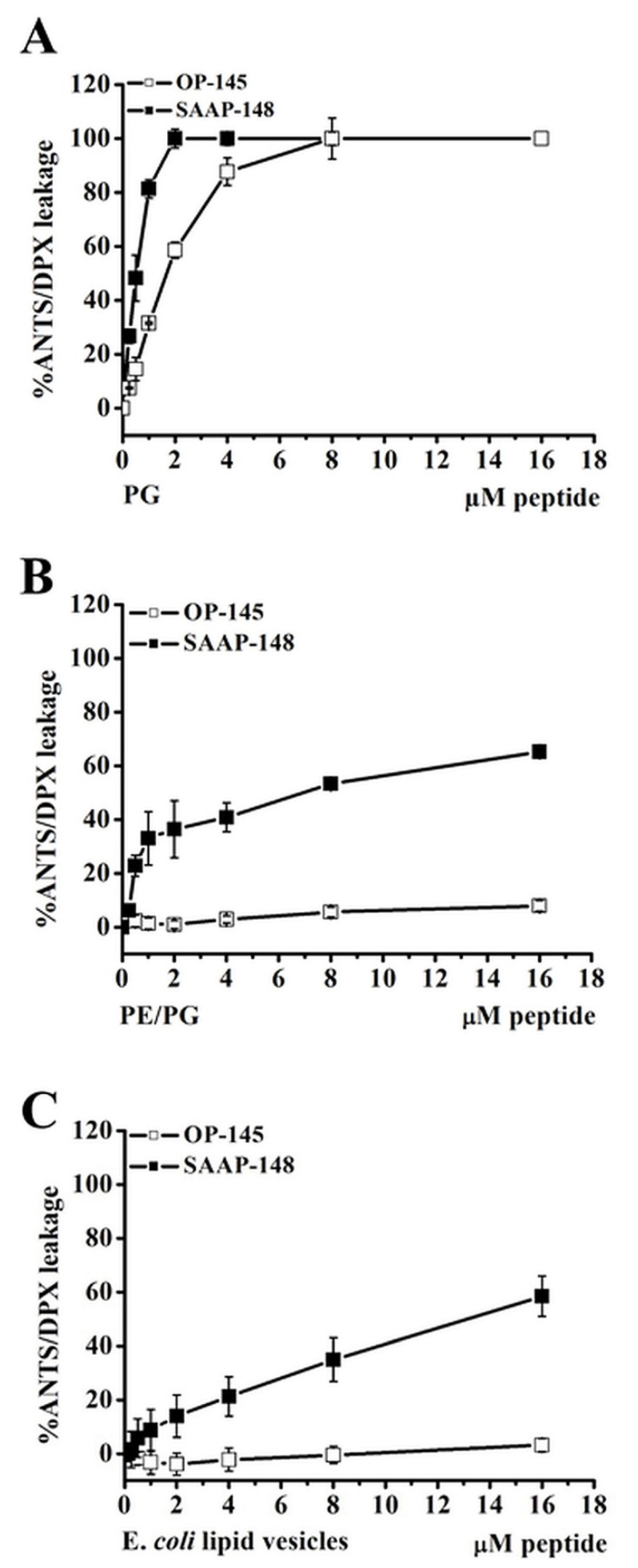
Leakage of membrane models PG (**A**), PE/PG, (**B**) and *E. coli* polar lipids (**C**) in absence and presence of OP-145 (squares) and SAAP-148 (circles) at indicated concentration ranging from 0.25 to 16 µM for membrane model which corresponds to lipid to peptide molar ratio of 200:1-3.125:1. Data points are averages ± standard deviations of three independent experiments.

In order to examine if electrostatic interactions significantly contribute to the membrane disruption potential of the peptides, we performed membrane permeabilization assays in analogy to zeta potential measurements. Disruption of model membranes upon titration of AMPs was tested by following leakage of the fluorescent dye ANTS from LUVs composed of PG, PE/PG, and *E. coli* polar lipids. Figure 6 shows the highest disruption potential of the peptides for PG membranes for both peptides. Most surprisingly, OP-145 did not induce leakage of membranes having higher zwitterionic content, PE/PG, and *E. coli* polar lipids. A significantly lower content (~30–50%) of the latter model membranes was released upon titration to SAAP-148. The reduced ability to disrupt the *E. coli* polar lipid membrane, especially lacking by OP-145, indicates that no direct phospholipid–peptide interaction is responsible for some degree of transmembrane effect observed for OP-145 on the *E. coli* cytoplasmic membrane (Figure 4A). Because this transmembrane effect occurred after the time OP-145 requires killing *E. coli*, we assume that the strong surface charge neutralization of *E. coli* during the killing process (Figure 3), e.g., effects induced on the outer membrane contribute to the destabilization of the cytoplasmic membrane. This effect cannot be observed in simple membrane model studies composed of solely phospholipids assembled in one single membrane. In respect of a complex membrane architecture of Gram-negative bacteria, the *E. coli* lipid model membrane resembles the lipid composition of the *E. coli* cytoplasmic membrane, but not of the outer membrane.

## 4. Discussion

The unspecific mode of action of AMP, the disruption of the membrane, and hence, the fundamental barrier function of a cell, can be seen as a mechanism that applies to a broad spectrum of bacterial cells. Although no direct consensus exists on which factors trigger AMPs preferentially towards Gram-positive or Gram-negative bacteria, the number of AMPs active against Gram-positive bacteria is larger [13]. This might be ruled by the high hydrophobicity of some peptides, which prevents their translocation through the outer membrane of Gram-negative bacteria [32]. Due to the different nature of the membranes, stronger forces are needed to disrupt (both) membranes in Gram-negative bacteria compared to a (single) membrane of Gram-positive bacteria. This is very often manifested by higher peptide demand for Gram-negative bacteria. Despite that, the negatively charged bacterial surface is exposed to attract such cationic molecules as AMPs. However, do all the peptides pass through the bacterial envelopes and further go to the cytoplasmic membrane? In many studies, it was shown that AMPs efficiently bind and interact with the anionic compounds on bacterial surfaces, e.g., LTA and LPS [13,18,20]. However, such studies encompass a direct measurement of a single molecule-to-molecule interaction and do not mimic the right situation in vivo as the density of distributed molecules is different and more complex in a network of bacterial cell envelopes. Therefore, it is not clear if binding to such compounds directly induces cell death or if the peptide is just sequestered by them and not available for the cytoplasmic membrane. Anyway, the affinity of the peptide for a specific molecule might widely contribute to its biological function, and mode of action and might affect the peptide’s concentration range necessary to kill the bacterium.

Nevertheless, the increased binding to the bacterial surfaces might be important for the attraction of the peptides to the bacterial surface (i) to increase the peptide local concentration; (ii) to neutralize the LPS/LTA layer; (iii) to enhance forces needed to disintegrate phospholipid assembly in the outer as well the inner membrane. OP-145 and SAAP-148 are both active against a large variety of pathogens; and although OP-145 has already managed to reach clinical trials [33], it is not as effective as SAAP-148 [8], which is one of the most potent antimicrobial peptides reported in the literature. However, both of them show good membrane disrupting activity and both are effective in the low µM range [8,19]. In the face of it all, in our study, SAAP-148 indeed killed *E. coli* as well as *E. hirae* at a significantly lower concentration than OP-145, which is in agreement with previously published reports [8,19]. It also neutralized the surface charge of *E. coli* at much lower concentrations which might be ascribed to its higher positive net charge. Surprisingly, both peptides did not neutralize the surface of *E. hirae*. A number of studies reported a high binding affinity of OP-145 to cell wall components of Gram-positive bacteria, e.g., LTA [18,20]. Given the fact that polyanionic LTA does not influence peptide activity on the membrane, it was assumed that electrostatic binding to cationic peptides serves as a ladder for peptides to traverse through the cell wall to the membrane. Our results led us to conclude that binding to the Gram-positive surface, as it is with *E. hirae*, is not electrostatically driven, but this does not exclude hydrophobic or other kinds of interactions of the peptides with LTA/TA. As teichoic molecules span through the entire cell wall, it is also a question if electrostatic interactions can be captured using zeta potential, which measures only changes to charges on the last surface layer.

Both peptides interact strongly with membrane phospholipids, but the effects induced at the molecular level are also slightly different as we already reported before for PG [8,19,20] and PG/CL [19] membranes. For the latter membranes, we did not observe electrostatic interaction while the membranes were disrupted. We concluded that a slightly larger hydrophobic patch in SAAP-148 might be responsible for deeper insertion into the membrane accompanied by stronger membrane permeabilization and disruption [8,19,20]. This also might hold true for *E. coli*, as SAAP-148 is able to permeabilize *E. coli* membranes and also disrupt membranes composed of *E. coli* polar lipids, which is not the case for OP-145. OP-145 neither permeabilizes *E. coli* cytoplasmic membranes nor membranes composed of extracted *E. coli* lipids. Given that the *E. coli* surface is completely neutralized by OP-145, it can be assumed that its mode of action more likely takes place on the outer membrane.

The fact, that the surface of all anionic membranes is neutralized by both peptides would at first glance point to the strong dependence on electrostatic interactions in disrupting the membrane. Indeed, our data also suggested that the more charged the bacterial surface is the more efficient membrane disruption can be achieved (Figure 5 and Figure 6). We observed 100% leakage of PG, a pure anionic membrane in the case of both peptides whereby less charged membranes were not sufficiently disrupted. Whereas SAAP-148 induced up to 25–30% leakage in PE/PG or membranes of *E. coli* lipid extracts, OP-145 did not permeabilize them. However, OP-145 also neutralized the surface of PE/PG and membranes of *E. coli* lipid extracts but was not able to disrupt them. We recently showed that the introduction of small amounts of also anionic CL in PG membranes was sufficient to reduce electrostatic interactions with both peptides [19]. This clearly points out that physico-chemical properties of phospholipids and their mixture as found in biological membranes largely control peptides activity. In respect of their physico-chemical properties, AMP- interactions with diverse phospholipids might be largely driven by three possible forces: (i) hydrophobic via fatty acyl tail interaction of any kind of PL (ii) electrostatic via binding to free phosphate group of PL as PG or CL and (iii) forming hydrogen bonds of free amine groups in the case of PE. For that reason, SAAP-148 might benefit from all three above-described forces as it is more charged and more hydrophobic than OP-145 and contains amino acids glutamine which with its side chain amine groups can form hydrogen bonds with PE. This latter might also contribute to more efficient permeabilization of PE-containing membranes by SAAP-148 than by OP-145. This may also explain why SAAP-148 is more efficient against *E. coli* than OP-145, as it is due to the high abundance of PE in the inner leaflet of the Gram-negative outer membrane SAAP-148 having more power to disrupt both *E. coli* membranes. Further, the presence of aromatic amino acid tryptophane might also enhance the activity of SAAP-148 as tryptophane has the largest driving force for membrane interface [34]. All these findings led us to conclude that increased capability for electrostatic interaction might also increase the membrane disruption potential of peptides, but other above-explained factors seem to be the driving force of their activity.

In summary, our results provide clear evidence: that charge-to-charge interaction between AMPs and bacteria is not the driving force of the membrane disruption activity in Gram-positive and Gram-negative bacteria. In Gram-positive bacteria, peptides neither neutralized the surface of *E. hirae* nor their model membranes [19] but showed increased membrane permeability during antimicrobial killing. In the case of Gram-negative bacteria, both peptides neutralized the surface of *E. coli* and model membranes but failed to induce marked membrane permeability in the concentration range where and when the peptides kill *E. coli*. Most importantly, neutralization of the *E. coli* surface by the peptides went hand in hand with antimicrobial killing. This led us to assume that their mode of action on Gram-negative bacteria involves electrostatic interactions, which are primarily important for actions on the outer membrane. Further, it seems that if not targeting other compounds the mechanism of action of OP-145 might primarily occur on the outer membrane as no direct membrane permeability of Gram-negative bacteria was observed. However, it is a question if this action is sufficient to kill bacteria and will require further studies in the future.

## 5. Conclusions

Based on our knowledge and our previous studies on these peptides [8,19,20], we propose the following mechanism for OP-145 and SAAP-148: The peptides attach to the Gram-positive bacterial surface and traverse through the cell wall to the cytoplasmic membrane. There, they insert their hydrophobic region into the fatty acyl chains thereby inducing “quasi” interdigitation or dimple formation. At the cellular level, such lipid disorder affects overall membrane integrity including membrane depolarization, changes in membrane fluidity, and increased membrane permeability. At least, this is manifested in membrane rupture and cell death.

On the other side, peptides immediately bind to negatively charged components and neutralize the surface of Gram-negative bacteria. Then, they insert into the fatty acyl chain region of the outer membrane, inducing higher outer membrane permeability. Consequently, this already impairs membrane stability and cellular viability. Further, they traverse to the cytoplasmic membrane and insert again into the fatty acyl chain region of the cytoplasmic membrane, whereby the membrane damage may not necessarily result in membrane rupture. The increased membrane permeability for small ions will be sufficient to result in cell death.

## Figures and Tables

**Table 1:** Antimicrobial activity of OP-145 and SAAP-148 against *E. coli* and *E. hirae*. Peptides were exposed either to 1 × 10^6^ CFU/mL of *E. coli* and *E. hirae* in presence of NaPi buffer or to 1 × 10^7^ CFU/mL of bacteria suspended in Hepes buffer. The 99.9% lethal concentration (LC99.9%) is defined as concentration necessary to kill 99.9% of the cells. The LC_99.9%_ are identical in all experiments, which have been performed independently at least three times.

	Antimicrobial Activity LC_99.9%_			
	(10^6^ CFU/mL, Napi)		(10^7^ CFU/mL, Hepes)	
Peptide	*E. coli*	*E. hirae*	*E. coli*	*E. hirae*
OP-145	6.4 µM	3.2 µM	>51.2 µM	51.2 µM
SAAP-148	1.6 µM	0.4 µM	6.4 µM	12.8 µM

## Data Availability

The data presented in this study are available on request from the corresponding author.
